# Gastric Duplication Cyst: A Report of a Rare Case

**DOI:** 10.7759/cureus.40285

**Published:** 2023-06-12

**Authors:** Loganathan Jayapal, Santhosh Kumar, Aravind Baskaran, TG Balachandar, Sudeepta K Swain

**Affiliations:** 1 Gastrointestinal Surgery & Liver Transplantation, Apollo Hospitals, Chennai, IND

**Keywords:** cross-sectional imaging, confirmatory histopathology, complete resection, adult, gastric duplication cyst

## Abstract

Gastric duplication cysts (GDCs) are rare congenital anomalies that primarily occur in childhood but can also manifest in adults. While the ileum is the most common site of duplication, gastric duplications are infrequent. Symptomatic GDCs typically present with upper abdominal pain, vomiting, and occasionally as palpable abdominal masses. Diagnostic imaging, particularly cross-sectional techniques, plays a crucial role in identifying these cysts, and surgical resection is the definitive curative treatment.

We report the case of a 44-year-old female who presented with severe right-side upper abdominal pain accompanied by non-bilious vomiting. Initial basic blood investigations yielded normal results. Subsequent contrast-enhanced computed tomography revealed a non-enhancing cystic lesion of size 9x8.5x6.5cm in the left suprarenal region lying posterior to the stomach suggestive of either a GDC or an adrenal cyst. Another hyperdense peripherally enhancing lesion was observed in the right adrenal gland, indicating a right adrenal cyst with internal hemorrhage. During laparotomy, the left side cystic lesion was found arising from the posterior wall of the greater curvature of the stomach, along with another cystic lesion of about 3x3cm originating from the right adrenal gland. Both cystic lesions were successfully excised, and the patient experienced a smooth postoperative recovery without any complications. Histopathological examination confirmed the presence of a cyst lined by gastric-type epithelium with underlying smooth muscle fibers consistent with GDCs. The right adrenal gland cystic lesion exhibited central areas of hemorrhage and necrosis.

## Introduction

Gastric duplication cysts (GDCs) are a rare congenital anomaly [1]. They usually occur in childhood and rarely in adults. The most common site of duplication is the ileum, although they can rarely occur in the stomach [2]. If symptomatic, they present with upper abdominal pain, vomiting, and rarely as a palpable abdominal mass. Cross-sectional imaging is diagnostic, and surgical resection is curative [3].

## Case presentation

**Figure 1:**
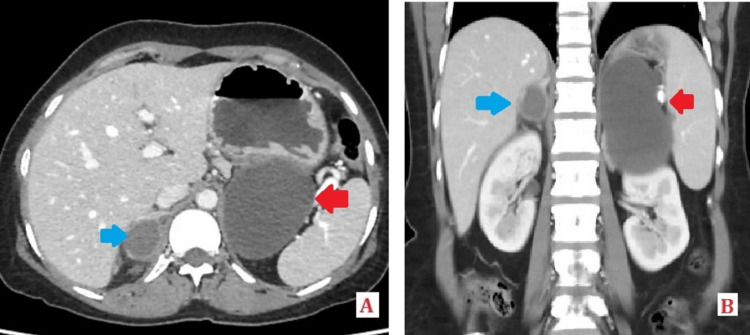
Axial view (A) and coronal view (B) of CECT abdomen with the red arrow showing the non-enhancing cystic lesion over the left adrenal gland region (gastric duplication cyst) and the blue arrow showing the hyperdense peripherally enhancing lesion in the right adrenal gland, suggestive of hemorrhage within the cyst. CECT:  Contrast-enhanced computed tomography

A 44-year-old female with no co-morbidities presented with severe right-side upper abdominal pain of continuous nature without any radiation, accompanied by scanty, non-bilious vomiting that lasted for two days. Her basic blood investigations and ultrasound of the abdomen were normal. Contrast-enhanced computed tomography (CECT) of the abdomen was performed, which revealed a non-enhancing cystic lesion of size 9x8.5x6.5cm over the left suprarenal region, abutting the left adrenal gland and posterior wall of the stomach. The cyst showed no internal septations, calcifications, or enhancing solid components. The adjacent fat planes were maintained, and no perilesional lymphadenopathy was observed. The differential diagnosis considered was adrenal cysts or GDCs. Additionally, a hyperdense peripherally enhancing lesion measuring 2.5x2x2.5cm was identified in the right adrenal gland, indicative of a right adrenal cyst with internal hemorrhage (Figures 1A, 1B).

**Figure 2:**
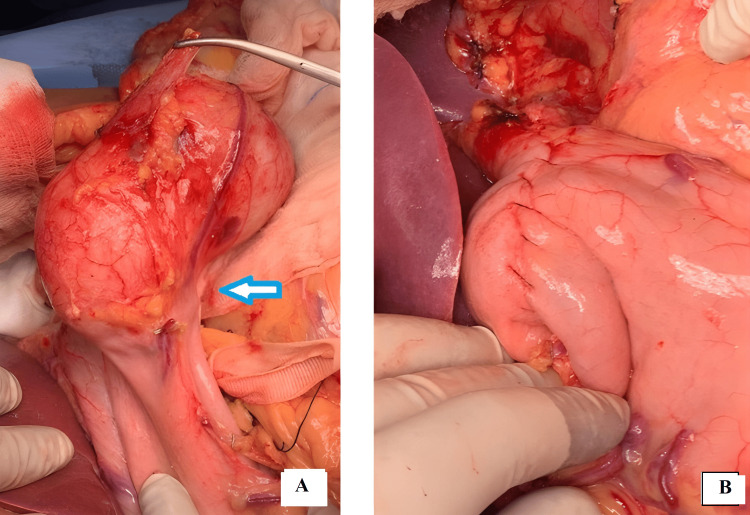
Intraoperative pictures (A) gastric duplication cyst showing the common wall shared (arrow) with stomach and (B) after excision and repair of the cyst

During laparotomy, a cystic lesion of approximately 10x10cm was found to arise from the posterior wall of the stomach along the greater curvature without any communication to the stomach, and another cystic lesion measuring approximately 3x3cm was found to originate from the right adrenal gland. Both the GDC and the right adrenal cyst with hemorrhage, which could have contributed to the abdominal pain, were successfully excised (Figures 2A, 2B). The patient had a smooth postoperative recovery without any complications. The final histopathological report confirmed the presence of a cyst lined by gastric-type epithelium with underlying smooth muscle fibers. A 5mm polyp was identified in the cyst wall, showing no dysplastic changes, consistent with a GDC. The cystic lesion in the right adrenal gland exhibited central areas of hemorrhage and necrosis.

## Discussion

GDC comprises 2-9% of all gastrointestinal duplication cysts, with a female preponderance (M: F = 1:8) [4]. Most diagnoses are made within the first three months of life [5]. Duplication cysts can be communicating or non-communicating with the gastrointestinal tract and may be cystic or tubular. The most common type (80%) is the non-communicating and cystic form [4,6]. Symptoms are often related to the cyst's location, such as recurrent abdominal pain, vomiting, difficulty in feeding, and a palpable abdominal mass. Complications include obstruction, bleeding, fistulation, pancreatitis, acute abdomen, and rarely malignant transformation, with adenocarcinoma being the most common type [7].

Several theories have been proposed about the formation of duplication cysts, including recanalization defects, split notochord, intestinal ischemia in early intrauterine life, and embryonic bands producing traction diverticula [8]. The essential features for diagnosis include 1) continuity of the cyst wall with the stomach, 2) smooth muscle layer of the cyst shared with the stomach, 3) the cyst has an epithelial lining of the gastrointestinal tract, and 4) the cyst and stomach share a common blood supply [9].

The cyst appears as a hypoechogenic lesion on ultrasonography. In the CECT abdomen, it presents as a round or tubular lesion with a thin and slightly enhancing wall. In endoscopic ultrasound, the lesion appears as an inner echogenic mucosal and outer hypoechoic muscle layer. The rate of misdiagnosis with CT scans ranges between 43% and 70%. MRI does not seem to improve diagnostic accuracy. Definitive diagnosis of GDCs depends on surgical findings and the final histopathology report [10].

Complete surgical resection with excision of the shared wall is the treatment of choice [11]. For malignant cases, surgical resection of the stomach and regional lymphadenectomy with oncologic principles, as described for carcinoma of the stomach, are recommended.

## Conclusions

GDCs are relatively uncommon gastrointestinal anomalies, representing a small percentage of all duplication cysts. Most diagnoses occur within the first three months of life and are rare in adults. This case report emphasizes the importance of maintaining high suspicion for diagnosing GDCs in adults, based on findings from cross-sectional imaging, and the subsequent decision for surgical resection. Surgical exploration and histopathological examination are necessary for confirming the definitive diagnosis of GDCs. The treatment of choice is complete surgical resection, which includes excision of the shared wall. Surgical resection of the stomach with regional lymphadenectomy, following oncologic principles similar to stomach carcinoma, is recommended in cases involving malignancy.

## References

[1] Theodosopoulos T, Marinis A, Karapanos K, Vassilikostas G, Dafnios N, Samanides L, Carvounis E (2007). Foregut duplication cysts of the stomach with respiratory epithelium. World J Gastroenterol.

[2] Seijo Ríos S, Lariño Noia J, Abdulkader Nallib I, Lozano León A, Vieites Pérez-Quintela B, Iglesias García J, Domínguez Muñoz JE (2008). Adult gastric duplication cyst: diagnosis by endoscopic ultrasound-guided fine-needle aspiration (EUS-FNA) (Article in Spanish). Rev Esp Enferm Dig.

[3] Perek A, Perek S, Kapan M, Göksoy E (2000). Gastric duplication cyst. Dig Surg.

[4] Scatizzi M, Calistri M, Feroci F (2005). Gastric duplication cyst in an adult: case report. In Vivo.

[5] Takazawa S, Uchida H, Kawashima H, Tanaka Y, Sato K, Jimbo T, Iwanaka T (2015). Laparoscopic partial gastrectomy of a huge gastric duplication cyst in an infant. Nagoya J Med Sci.

[6] Menon P, Rao KL, Saxena AK (2004). Duplication cyst of the stomach presenting as hemoptysis. Eur J Pediatr Surg.

[7] Stephen TC, Bendon RW, Nagaraj HS, Sachdeva R (1998). Antral duplication cyst: a cause of hypergastrinemia, recurrent peptic ulceration, and hemorrhage. J Pediatr Gastroenterol Nutr.

[8] Stern LE, Warner BW (2000). Gastrointestinal duplications. Semin Pediatr Surg.

[9] Rowling JT (1959). Some observations on gastric cysts. Br J Surg.

[10] Macpherson RI (1993). Gastrointestinal tract duplications: clinical, pathologic, etiologic, and radiologic considerations. Radiographics.

[11] Ildstad ST, Tollerud DJ, Weiss RG, Ryan DP, McGowan MA, Martin LW (1988). Duplications of the alimentary tract. Clinical characteristics, preferred treatment, and associated malformations. Ann Surg.

